# Weighing up the potential of “superfoods” compounds of green tea or turmeric as adjuncts in comparison to established therapeutical approaches for periodontal disease

**DOI:** 10.1007/s00784-024-06122-2

**Published:** 2025-01-14

**Authors:** Georg Heuzeroth, Manuela Elena Kaufmann, Isabelle Herter-Aeberli, Ulrich Schlagenauf, Chun Ching Liu, Spyridon N. Papageorgiou, Patrick R. Schmidlin

**Affiliations:** 1 Clinic of Orthodontics and Pediatric Dentistry, Center for Dental Medicine, University of Zurich, Plattenstrasse 11, Zurich, 8032 Switzerland; 2 Clinic of Conservative and Preventive Dentistry, Center for Dental Medicine, University of Zurich, Zurich, Switzerland; 3 Laboratory of Nutrition and Metabolic Epigenetics, Department of Health Sciences and Technology, ETH Zurich, ETH Zurich, Zurich, Switzerland

**Keywords:** Superfoods, Periodontitis, Gingivitis, Periodontal disease, Randomized trial, Systematic review

## Abstract

**Objective:**

Aim of this study was to critically appraise clinical evidence on the potential benefits of adjunctive use of superfoods green tea and turmeric as mouthrinse or local delivery agents in the treatment of periodontal disease.

**Materials and methods:**

Electronic searches were performed in four databases for randomized trials from inception to February 2024 assessing the supplemental use of superfoods green tea and turmeric for gingivitis/periodontitis treatment. After duplicate study selection, data extraction, and risk-of-bias assessment with the RoB 2 tool, random-effects meta-analyses of Mean Differences (MD) or Standardized Mean Differences (SMD) with their 95% confidence intervals (CI) were performed.

**Results:**

Nineteen studies (814 patients) were included, with 11 on gingivitis and 8 on periodontitis patients. No benefits were seen from the use of mouthwashes containing green tea extract or turmeric for gingivitis treatment, while green tea was associated with smaller Gingival Index (GI) reductions than chlorhexidine (5 studies; MD = 0.08; 95% CI = 0.01 to 0.14; *P* = 0.01). As far as periodontitis treatment is concerned, local supplementation with turmeric showed no benefits, whereas local supplementation with green tea extract was associated with improved treatment outcomes in terms of probing depth (4 studies; MD=-0.79; 95% CI=-1.29 to -0.29 mm; *P* = 0.002) and GI (3 studies; MD=-0.53; 95% CI=-1.01 to -0.05; *P* = 0.02) than the control group. However, the strength of evidence was moderate to very low due to bias, imprecision, and inconsistency.

**Conclusion:**

Limited evidence indicates that supplemental use of green tea extract is associated with improved periodontal treatment outcomes. However, the strength of evidence is weak and further research is needed.

**Clinical relevance:**

Green tea extract could be a natural adjunct to enhance periodontal treatment, without the potential side-effects of other adjuncts like chlorhexidine.

**Supplementary Information:**

The online version contains supplementary material available at 10.1007/s00784-024-06122-2.

## Introduction

Periodontal disease, characterized by gingival inflammation, formation of gingival pockets, progressive attachment loss and bone loss [1], is estimated to affect a considerable portion of the world population with varying burden among different regions and populations [2]. While bacterial plaque serves as the primary etiological factor in the development of gingivitis, which can progress to periodontitis, it is crucial to acknowledge that periodontal health is influenced by many factors, including genetic predisposition, systemic health, and nutritional status, which are recognized as key contributors [3]. In the context of periodontal destruction, an intricate interplay emerges between the plaque biofilm and the host’s immunoinflammatory response, resulting in an imbalance between bacterial activity and the host’s defense mechanisms [4].

When it comes to the treatment of periodontal disease, the initial therapeutic approach entails scaling and root planing (SRP) and has attained a prominent position as the standard treatment modality [1]. Research in adjuncts for periodontal therapy has focused mainly on the use of antibiotics or antiseptics, both systemic and locally delivered, which has demonstrated notable efficacy in diminishing periodontal probing depths and thereby facilitating the restoration of clinical attachment levels and mitigation of the likelihood for subsequent attachment loss [5, 6]. Even though management of periodontitis entails mainly the removal of causal factors, the scope of possible factors that influence progression of tissue destruction or improve the outcome of therapy is large. The association between nutrition and periodontal health has been investigated in many studies, linking greater intakes of foods and nutrients with antioxidant and anti-inflammatory activity to a reduced risk of developing periodontal disease and improved periodontal healing [7].

The potential benefits of so-called superfoods for tissues / processes also involved in periodontal health have gained considerable attention the last years. These include, among others, natural herbal products like chia seeds, quinoa, spirulina, turmeric, acai-berry, propolis, aloe vera, green tea, cranberry, calendula, myrrha and salvia [8–10]. However, there is a lack of clear criteria for defining what qualifies as a superfood and which of them can be supported by ample clinical experimental evidence, in terms of better outcomes of periodontal treatment. Therefore, the present review aimed to evaluate the efficacy of the administration of “superfood” agents in patients who underwent non-surgical periodontal therapy, as compared to established treatment approaches and adjuncts. The initial focused question for this review was: is supplemental administration of specific superfoods associated with improved outcomes in the non-surgical treatment of periodontal disease (gingivitis or periodontitis) among human patients?

## Materials and methods

This review was conducted and reported in accordance with the Cochrane Handbook [11] and the PRISMA (Preferred Reporting Items for Systematic Reviews and Meta-Analyses) statement for systematic reviews [12], respectively.

### Eligibility criteria

Included in this review were Randomized Clinical Trials (RCT) in humans of either parallel or within-person (split-mouth) randomization (i.e. local delivery of different agents at different sites within the mouth of the same patient), where one or more ‘superfoods’ were used as adjuncts in the treatment of periodontal disease (gingivitis or periodontitis). Superfoods should be used (alone or in combination with standard care) with a specific protocol and compared to a passive control group (no treatment, standard care or placebo) or an active control group consisting of chlorhexidine-containing products. Due to logistic constraints, included were only studies in English or German. Excluded were non-randomized studies, non-clinical studies, animal studies, in vitro studies, studies including patients with systemic diseases, and studies administering only isolated components or parts of a superfood.

### Information sources and search strategy

A literature search was performed in MEDLINE through PubMed, Embase, Biosis Previews Database, and the Cochrane Library, using a separate search for each superfood and with the search covering from inception of each database to February 19th, 2024. Search terms consisted of the superfood combined with the keyword periodontitis and additionally with the MeSH terms antimicrobial and/or anti-inflammatory: (superfood) AND (“periodontitis”) AND (“anti-microbial” OR “anti-inflammatory”). The list of all superfoods that were used in the separate searches can be found in Appendix 2.

### Selection process

Two authors (GH, MEK) independently screened titles and abstracts of the electronic search and assessed them in a first step for possible inclusion in the review, followed by assessment of their full text. Any disagreements between them were resolved by discussion with a third author (PRS). Included were only superfoods that were assessed by at least three studies in total, in order to provide more robust information.

### Data collection process and data items

Data extraction was performed in duplicate by two independent authors (GH, MEK), stratified for gingivitis and periodontitis patients, and included: (i) study characteristics (design, setting, country); (ii) patient demographics (number, sex, age); (iii) superfood administered, including administration mode (mouthwash or local delivery); (iv) administration dosage and protocol; (v) control group used; (vi) duration of follow-up; and (vii) outcomes assessed. As studies on both gingivitis and periodontitis patients were included the Gingival Index (GI) [13] was set as the primary outcome, due to its relevance to both diseases. Secondary outcomes included the probing depth and any forms of the Plaque Index (PI), including among others those by Silness and Loe [14] or others [15–17].

### Risk of bias

The risk of bias of all included studies was assessed with the Cochrane collaboration’s RoB 2 tool for randomized trials [18] by two independent authors (GH, MEK) and all disagreements were resolved again by discussion with a third person (PRS). The RoB 2 tool assesses the possible impact of bias (i) arising from the randomization process, (ii) due to deviations from intended interventions, (iii) due to missing outcome data, (iv) in measurement of the outcome, and (v) selection of the reported result.

### Synthesis methods

Pairwise meta-analyses were used to synthesize data from two studies or more assessing the same comparison. The Mean Difference (MD) with its 95% Confidence Interval (CI) was chosen as effect measure for GI and probing depth, while the Standardized Mean Difference (SMD; Cohen’s d) was used to combine different PIs. As the effect of periodontal treatment was expected to vary according to several factors (including genetic predisposition, baseline severity, patient compliance, and individual biological response), a random-effects model with a restricted maximum likelihood variance estimator [19] was deemed a priori more appropriate to capture this variability and calculate the average distribution of associations across studies [20]. Between-study heterogeneity was assessed through visual inspection of contour-enhanced forest plots (Appendix 1) [21] and through estimation of tau^2^ (absolute heterogeneity) or I^2^ (relative inconsistency) with their uncertainty intervals. 95% predictions were calculated, which incorporate identified heterogeneity and assist in the interpretation of the meta-analytical estimates by providing a range of expected effects across various future clinical settings [22]. Random-effects subgroup analysis was used to assess differences between different administration modes (mouthwash or local delivery) for meta-analyses with at least 5 studies. Sensitivity analysis was performed for meta-analyses with ≥ 5 studies according to the study’s risk of bias. A sensitivity analysis was performed comparing the effects of active agents between reference groups of either control / placebo or standard treatment (Appendix 1). Analyses of reporting biases (including small-study effects and the possibility of publication bias) were planned to be assessed for meta-analyses with ≥ 10 studies (Appendix 1), but no such meta-analyses were ultimately performed. The Grades of Recommendations, Assessment, Development, and Evaluation (GRADE) approach was employed [23] to gauge the certainty around the results of meta-analyses and findings were presented with a revised summary of findings Table [24]. All analyses were conducted in R 4.2.2. (R Foundation for Statistical Computing, Vienna, Austria) by one author (SNP), with open dataset [25], alpha set at 0.05 (except for between-subgroups where it was 0.10) and a two-sided P-value.

## Results

### Study selection

**Fig. 1:**
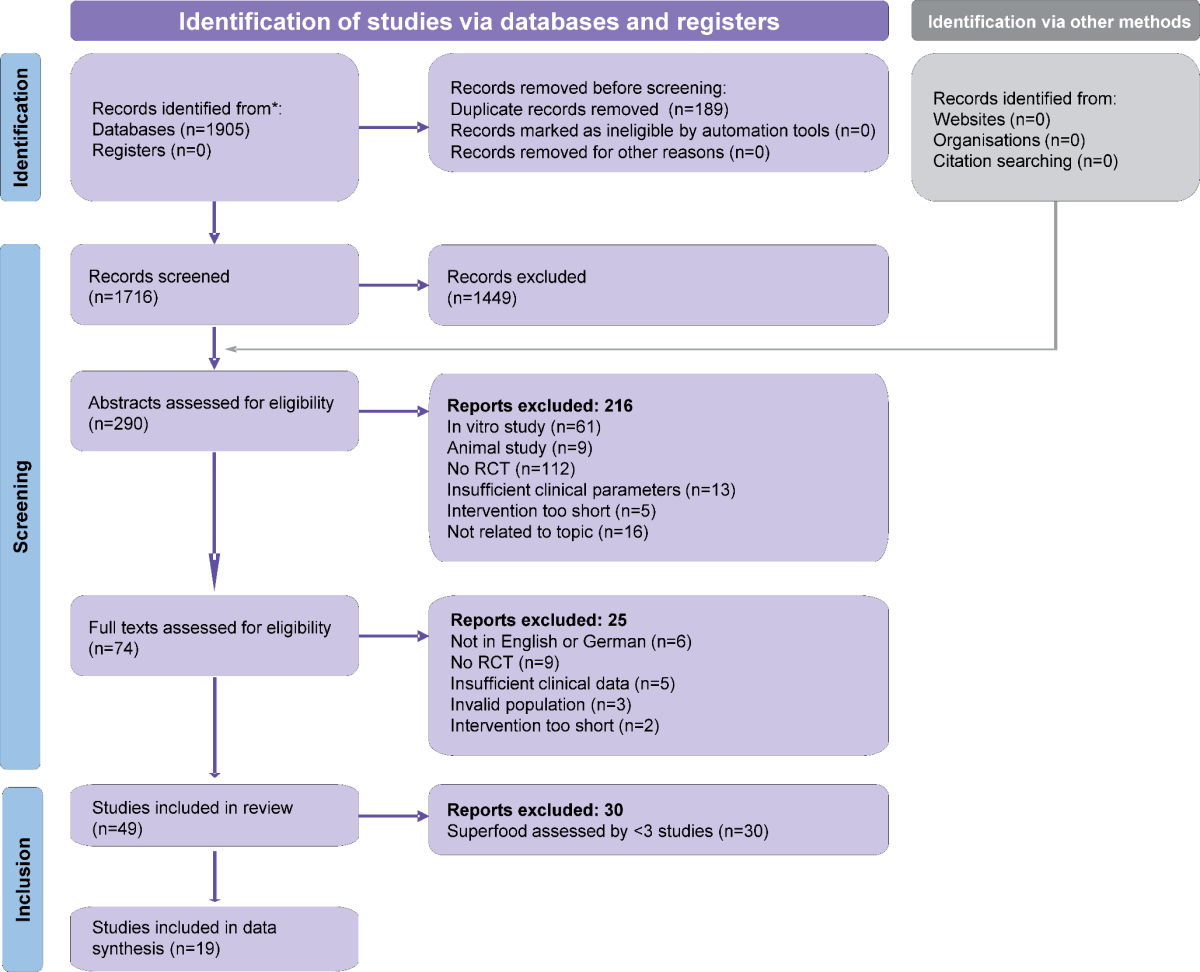
PRISMA flowdiagram for the identification and selection of eligible studies. RCT, randomized clinical trial

From 1905 records identified from the electronic search, 1449 articles were excluded by title (Fig. 1). Another 216 records were excluded by screening their abstracts, while a total of 74 full texts were assessed for eligibility (Appendix 2). Initially, the present study aimed to assess the effect of several superfood-based agents used adjunctly as mouthrinse or locally in the treatment of periodontal disease. However, from the 49 potentially eligible remaining studies, 30 studies were excluded as many superfood-based agents were used by less than 3 studies and precluded basic quantitative synthesis (meta-analysis). As such, the review’s scope was adapted post hoc to focus solely on green tea- or turmeric-containing agents used as adjuncts for the treatment of periodontal disease, and a total of 19 studies were ultimately included.

### Study characteristics

Details of the included studies are given in Table 1, with ten of them assessing gingivitis patients and eight of them assessing periodontitis patients. From the ten studies on gingivitis, all of them were of parallel design and were conducted in a university clinic. The majority (*n* = 8; 80%) of them were conducted in India, while one study was conducted in Iran and one in the United States. The average sample size was 48.2 patients/study, while patient sex was specified only in three (30%) of the studies (with 39.3% of patients being male). Six of the studies prescribed to the patients a mouthwash with green tea extract and four prescribed a turmeric mouthwash. Superfoods were most compared to a chlorhexidine mouthwash (*n* = 9; 90%) and in two instances (20%) to a control / standard care group. Follow-up ranged from 1 to 12 weeks and all studies assessed both some kind of PI and GI.

**Table 1:** Characteristics of included studies

Author	Design; setting; country†	Patients (M/F); age*	Intervention	Dosage; protocol	Control	Follow up (wks)	Outcomes	
**Gingivitis patients**								
Andhare 2022	RCT_PAR_; Uni; IND	45 (NR); (18.0–40.0)	Green tea; MW	0.5% solution; 10 ml for 1’ 2x/d	G1: SRP G2: CHX 0.2%	2; 3	PI_SL_; GI	
Balappanavar 2013	RCT_PAR_; Uni; IND	30 (15/15); 20.9	Green tea; MW	0.5% solution; 30s rinsing after brushing; 3x/d	CHX 0.2%	1; 2; 3	PI_SL_; GI	
Deshpande 2021	RCT_PAR_; Uni; IND	60 (41/19); (10.0–14.0)	Green tea; MW	5% solution; 2x/d	CHX 0.2%	2; 4	PI_SL_; GI	
Divya 2017	RCT_PAR_; Uni; IND	60 (NR); (15.0-NR)	Turmeric; MW	0.1% extract; peppermint oil flavour, chloroform water, water; 10mL for 1 min 2x/d for 21d	CHX 0.2%	3	PI_SL_; GI	
Lauten 2005	RCT_PAR_; Uni; USA	17 (3/14); (18.0-NR)	Green tea; MW	Solution with melaleuca oil, manuka oil, calendula flower extract, liquid extract, and ethanol; 30s rinsing; 2x/d	Control: water	6; 12	PI_QH_; GI	
Mali 2012	RCT_PAR_; Uni; IND	60 (NR); (15.0-NR)	Turmeric; MW	0.1% extract; 10mL for 1 min 2x/d for 21d 30’ after brushing	CHX 0.2%	2; 3	PI_QHTGG_; GI	
Priya 2017	RCT_PAR_; Uni; IND	30 (NR); (18.0–24.0)	Green tea; MW	0.25% solution; 30’ after tooth brushing; protocol unspecified	CHX 0.12%	2; 4	PI_QHTGG_; GI	
Sarkar 2023	RCT_PAR_; Uni; IND	20 (NR); (18.0–40.0)	Turmeric; MW	10 ml	CHX 0.12%	2; 3; 4	PI_SL_; GI	
Sharma 2023	RCT_PAR_; Uni; IND	90 (14/76); (18.0–60.0)	Green tea; MW	2 g green tea extract, 4 mL Tween 80 oil, 0.3 mL peppermint oil, 150 mg methyl paraben, 5 g mannitol, and 500 mg poloxamer for every 100 ml; 10 ml, 2x/d.	G1: control (saline) G2: CHX 0.2%	3	PI_QHTGG_	
Waghmare 2011	RCT_PAR_; Uni; IND	100 (NR); (25.0–35.0)	Turmeric; MW	0.1% extract; 0.005% peppermint oil, pH adjusted to 4, gargling 2x/d; 10mL in 1:1 with water after brushing for 21d	CHX 0.2%	2; 3	PI_QHTGG_; GI	
Yaghini 2019	RCT_PAR_; Uni; IRN	60 (24/36); (20.0–50.0)	Green tea; MW	50 drops of tea-aloe vera solution to half a glass of water; 1 min rinsing; 2x/d	CHX 0.2%	2	PI_SL_; GI	
***Periodontitis patients***								
Anuradha 2015	RCT_SM_; Uni; IND	30 (NR); (25.0–60.0)	Turmeric; LOC	1% gel applied in the periodontal pocket and covered with Perio-Pak	SRP	4; 6	PI_QHTGG_; GI; PD	
Chava 2013	RCT_SM_; Uni; IND	30 (15/15); 38.9	Green tea; LOC	1.5% gel in the periodontal pocket; 1x at 0d	Placebo	4	GI; PD	
Guru 2020	RCT_PAR_; Uni; IND	45 (36/9); 39.5	Turmeric; LOC	Pluronic F-127 gel and 2% extract in the periodontal pocket and covered with Perio-Pak	G1: SRP G2: CHX 1.0%	3; 6	PI_SL_; GI; PD	
Hirasawa 2002	RCT_SM_; Uni; JPN	6 (3/3); (41.0–64.0)	Green tea; LOC	5% dissolved with ethanol and lyophilized	G1: Control (no SRP) G2: Placebo	8	PD	
Kaur 2019	RCT_PAR_; Uni; IND	29 (20/9); (20.0–65.0)	Turmeric; LOC	1% gel; single application subgingivally after SRP + Perio-Pak	SRP	4; 12	PI_SL_; PD	
Nagate 2020	RCT_SM_; Uni; IND	20 (NR); (20.0–40.0)	Green tea; LOC	Pluronic F-127 gel and extract	SRP	4	PI_SL_; GI; PD	
Rattanasuwan 2016	RCT_PAR_; Uni; THA	42 (22/20); 50.2	Green tea; LOC	12% gel (extract, water, alcohol, sugar alcohols, parabens, cellulose) in the periodontal pocket; 1x at 0d, 7d, 14d	Placebo	1; 2; 6.3; 14.9; 27.7	GI; PD	
Singh 2018	RCT_SM_; Uni; IND	40 (22/18); 34.0	Turmeric; LOC	Chip	G1: Control (SRP) G2: CHX 2.5% chip	4; 12	PI_SL_; GI; PD	

CHX, chlorhexidine; d, day; G, group; LOC, local delivery; M/F, male/female; MW, mouthwash; NR, not reported; PAR, parallel; PD, probing depth; QH, Quigley-Hein index; RCT, randomized clinical trial; s, second; SL, Silness and Löe index; SM, split-mouth; SRP, scaling and root planning; TGG, Turesky-G-G modification; Uni, university clinic; wks, weeks

* reported either as mean (one value) or, if mean not reported, as range (two values in parenthesis). † given their ISO-3 country codes

From the eight studies on periodontitis, the majority (*n* = 5; 63%) employed a split-mouth design, all were conducted in university clinics, and the majority (*n* = 6; 75%) originated from India. The average sample size was 30.3 patients/study and patient sex was reported in most (*n* = 6; 75%) of studies (and 59.6% of patients being male). Half of the studies supplemented periodontitis treatment with green tea and the other half with turmeric—both administered locally. In all instances, superfoods supplementation was compared to standard care / placebo, while chlorhexidine control groups were used in two studies. Follow-up ranged from 4 to 28 weeks and probing depth was the most consistently reported outcome (in all studies).

### Risk of bias in studies

The risk of bias of included trials with the RoB 2 tool is seen in Table 2 and Appendix 5. Seven studies were found to be in low risk of bias, 2 studies showed some concerns, and the remaining 6 studies were in high risk of bias. The most problematic domains (at least to some extent) were bias due to deviations from the intended interventions (9 studies; 47%), followed by bias in measurement of the outcome (7 studies; 37%), and bias arising from the randomization process (1 study; 5%).

**Table 2:** Detailed risk of bias assessment of included randomized trials with the Cochrane ROB 2 tool

Domain	1	2	3	4	5	
RoB…	Arising from the randomization process	Due to deviations from the intended interventions	Due to missing outcome data	In measurement of the outcome	In selection of the reported result	Overall
Andhare 2022	**Low**	**Low**	**Low**	**Low**	**Low**	**Low**
Anuradha 2015	**Low**	*High*	**Low**	*High*	**Low**	*High*
Balappanavar 2013	**Low**	***Some concerns***	**Low**	**Low**	**Low**	**Low**
Chava 2013	**Low**	***Some concerns***	**Low**	***Some concerns***	**Low**	*High*
Deshpande 2021	**Low**	**Low**	**Low**	**Low**	**Low**	**Low**
Divya 2017	**Low**	**Low**	**Low**	**Low**	**Low**	**Low**
Guru 2020	**Low**	***Some concerns***	**Low**	**Low**	**Low**	***Some concerns***
Hirasawa 2002	**Low**	***Some concerns***	**Low**	*High*	**Low**	*High*
Kaur 2019	**Low**	**Low**	**Low**	**Low**	**Low**	**Low**
Lauten 2005	**Low**	**Low**	**Low**	**Low**	**Low**	**Low**
Mali 2012	***Some concerns***	***Some concerns***	**Low**	*High*	**Low**	*High*
Nagate 2020	**Low**	***Some concerns***	**Low**	*High*	**Low**	*High*
Priya 2017	**Low**	**Low**	**Low**	**Low**	**Low**	**Low**
Rattanasuwan 2016	**Low**	**Low**	**Low**	**Low**	**Low**	**Low**
Sarkar 2023	**Low**	**Low**	**Low**	***Some concerns***	**Low**	**Low**
Sharma 2023	**Low**	**Low**	**Low**	**Low**	**Low**	**Low**
Singh 2018	**Low**	*High*	**Low**	***Some concerns***	**Low**	*High*
Waghmare 2011	**Low**	***Some concerns***	**Low**	**Low**	**Low**	***Some concerns***
Yaghini 2019	**Low**	**Low**	**Low**	**Low**	**Low**	**Low**

RoB, risk of bias

### Results of individual studies and data syntheses

The results of all individual included studies can be found in the openly provided dataset of this review [25], while meta-analyses were used to combine data from ≥3 similar studies, separately for gingivitis (Table 3) and periodontitis patients (Table 4).

**Table 3:** Performed meta-analyses on gingivitis patients

Intervention	Control	Outcome	*n*	Effect (95% CI)	*P*	tau^2^ (95% CI)	I^2^ (95% CI)	95% prediction
Green tea	Control / placebo	Plaque index_SL/QH_	4	SMD − 1.90 (-4.05, 0.25)	0.08	4.59 (1.32, 67.33)	95% (91%, 98%)	-12.26, 8.46
Green tea	Control / placebo	Gingival index	3	MD -0.26 (-0.56, 0.04)	0.09	0.06 (0.01, 3.09)	83% (48%, 94%)	-3.94, 3.43
Green tea	Chlorhexidine	Plaque index_SL/QH_	6	SMD − 0.40 (-0.88, 0.09)	0.10	0.23 (0.01, 2.39)	64% (13%, 85%)	-1.89, 1.10
Green tea	Chlorhexidine	Gingival index	5	MD 0.08 (0.01, 0.14)	0.01	0 (0, 0.01)	0% (0%, 79%)	-0.02, 0.17
Turmeric	Chlorhexidine	Plaque index_SL/QH_	4	SMD 0.28 (-0.53, 1.10)	0.50	0.60 (0.14, 9.09)	90% (78%, 96%)	-3.50, 4.06
Turmeric	Chlorhexidine	Gingival index	4	MD -0.03 (-0.10, 0.04)	0.42	0 (0, 0)	0% (0%, 85%)	-0.18, 0.12

CI, confidence interval; QH, Quigley-Hein index; MD, mean difference; n, studies; SL, Silness and Löe index; SMD, standardized mean difference

**Table 4:** Performed meta-analyses on periodontitis patients

Intervention	Control	Outcome	*n*	Effect (95% CI)	*P*	tau^2^ (95% CI)	I^2^ (95% CI)	95% prediction
Green tea	Control / placebo	Plaque index_SL_	1	MD -0.85 (-1.08, -0.62)	< 0.001	-	-	-
Green tea	Control / placebo	Gingival index	3	MD -0.53 (-1.01, -0.05)	0.02	0.16 (0.03, 6.52)	91% (75%, 96%)	-6.42, 5.36
Green tea	Control / placebo	Probing depth	4	MD -0.79 (-1.29, -0.29)	0.002	0.17 (0, 4.07)	69% (11%, 89%)	-2.89, 1.30
Turmeric	Control	Plaque index_SL/QH_	4	SMD − 0.49 (-1.74, 0.75)	0.44	1.52 (0.42, 22.79)	94% (87%, 97%)	-6.47, 5.48
Turmeric	Control	Gingival index	3	MD -0.23 (-0.63, 0.17)	0.25	0.12 (0.03, 4.55)	96% (92%, 98%)	-5.26, 4.80
Turmeric	Control	Probing depth	4	MD -0.28 (-0.77, 0.21)	0.26	0.22 (0.05, 3.26)	95% (89%, 97%)	-2.57, 2.01
Turmeric	Chlorhexidine	Plaque index_SL_	2	MD 0.07 (-0.09, 0.23)	0.36	0 (-)	15% (-)	-
Turmeric	Chlorhexidine	Gingival index	2	MD 0.02 (-0.11, 0.15)	0.78	0 (-)	0% (-)	-
Turmeric	Chlorhexidine	Probing depth	2	MD 0.14 (-0.06, 0.34)	0.17	0 (-)	0% (-)	-

CI, confidence interval; QH, Quigley-Hein index; MD, mean difference; n, studies; SL, Silness and Löe index; SMD, standardized mean difference

**Fig. 2:**
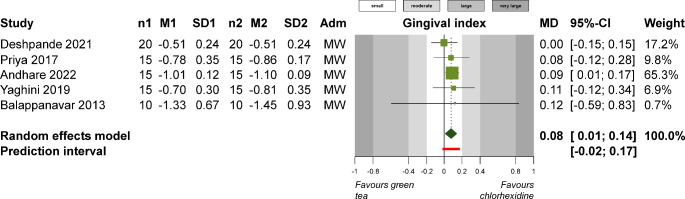
Meta-analysis on the effect of green tea extract supplementation versus chlorhexidine for the treatment of gingivitis in terms of gingival index. Adm, administration mode; MD, mean difference; CI, confidence interval; MW, mouthwash; n1/2, patients in experimental / control group; M1/2, mean in experimental / control group; SD1/2, standard deviation in experimental / control group

As far as gingivitis is concerned, green tea mouthwash showed no statistically significant improvement compared to control / placebo in terms of PI (3 studies; SMD − 0.93; 95% CI -2.33 to 0.47; *P* = 0.19; Appendix 6) or GI (3 studies; MD -0.26; 95% CI -0.56 to 0.04; Appendix 7). Compared to a chlorhexidine mouthwash, green tea mouthwash showed no difference in terms of PI (5 studies; SMD − 0.28; 95% CI -0.82 to 0.26; *P* = 0.31; Appendix 8) but showed significantly smaller GI reduction (5 studies; MD 0.08; 95% CI 0.01 to 0.14; *P* = 0.01; Fig. 2). The use of a turmeric mouthwash was not associated with significant differences compared to a chlorhexidine mouthwash in terms of PI (4 studies; SMD 0.28; 95% CI -0.53 to 1.10; *P* = 0.50; Appendix 9) or GI (4 studies; MD -0.03; 95% CI -0.10 to 0.04; *P* = 0.42; Appendix 10).

**Fig. 3:**
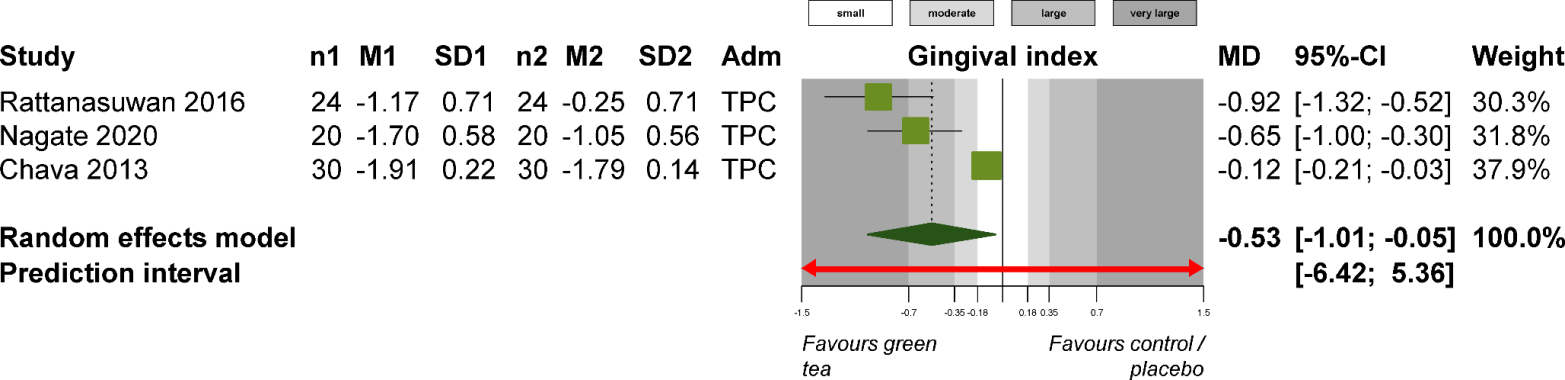
Meta-analysis on the effect of green tea extract supplementation versus chlorhexidine for the treatment of periodontitis in terms of gingival index. Adm, administration mode; CI, confidence interval; M1/2, mean in experimental / control group; MD, mean difference; n1/2, patients in experimental / control group; SD1/2, standard deviation in experimental / control group; TPC, topical administration

**Fig. 4:**
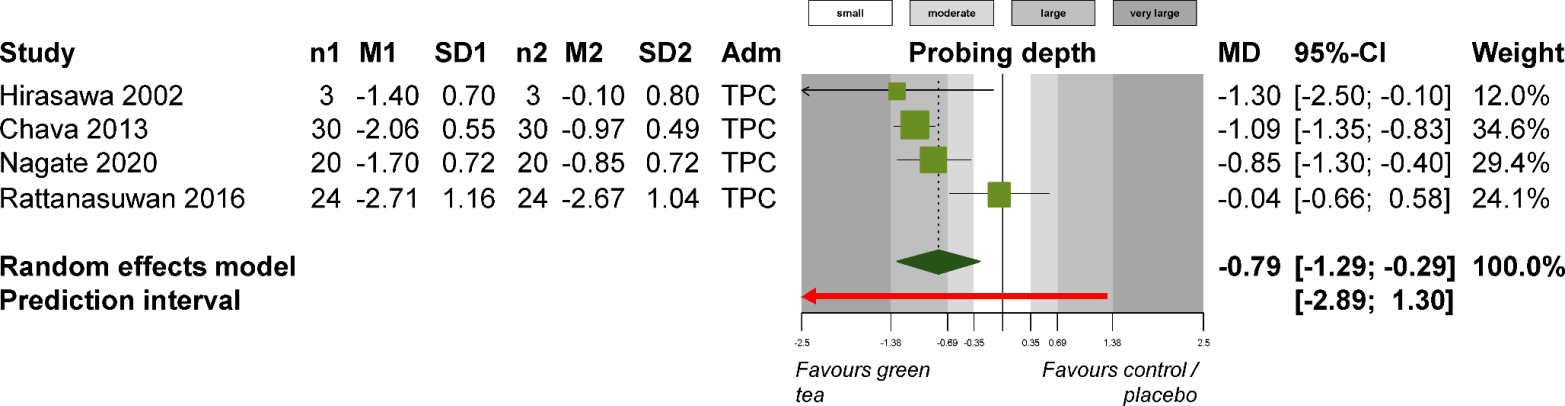
Meta-analysis on the effect of green tea extract supplementation versus chlorhexidine for the treatment of periodontitis in terms of probing depth. Adm, administration mode; CI, confidence interval; M1/2, mean in experimental / control group; MD, mean difference; n1/2, patients in experimental / control group; SD1/2, standard deviation in experimental / control group; TPC, topical administration

For periodontitis patients, supplementation with local delivery of green tea extract was associated with greater reductions in PI (1 study; MD -0.85; 95% CI -1.08 to -0.62; *P* < 0.001), GI (3 studies; MD -0.53; 95% CI -1.01 to -0.05; *P* = 0.02; Fig. 3), and probing depth (4 studies; MD -0.79 mm; 95% CI -1.29 to -0.29 mm; *P* = 0.002; Fig. 4) compared to standard periodontal treatment. On the other side, turmeric supplementation did not show any significant improvements to standard periodontal treatment in terms of PI (4 studies; SMD − 0.49; 95% CI -1.74 to 0.75; *P* = 0.44; Appendix 11), GI (3 studies; MD -0.23; 95% CI -0.63 to 0.17; *P* = 0.25; Appendix 12), or probing depth (4 studies; MD -0.28 mm; 95% CI -0.77 to 0.21 mm; *P* = 0.26; Appendix 13). Similarly, turmeric supplementation was not associated with any differences compared to chlorhexidine use in terms of PI (2 studies; MD 0.07; 95% CI -0.09 to 0.23; *P* = 0.36; Appendix 14), GI (2 studies; MD 0.02; 95% CI -0.11 to 0.15; *P* = 0.78; Appendix 15), or probing depth (2 studies; MD 0.14; 95% CI -0.06 to 0.34; *P* = 0.17; Appendix 16).

### Additional analyses

Subgroup analyses were planned to be performed according to administration mode for meta-analyses of ≥ 5 studies. This was the case for only two meta-analyses comparing green tea mouthwash to chlorhexidine mouthwash and assessing PI and GI (Table 3). However, as only mouthwash was used in all instances, no such subgroup analysis was possible.

Additionally, sensitivity analysis according to the risk of bias could not be performed for the same two meta-analyses, since all included studies in these were found to be in low risk of bias. Sensitivity analysis of meta-analyses on the absolute effect of green tea mouthwash did not find significant difference whether this was compared to control / placebo groups or standard treatment (scaling and root planning) (Appendix 17).

The strength of meta-evidence was assessed with the GRADE approach for meta-analyses on gingivitis (Table 5) and periodontitis patients (Table 6). For the treatment of gingivitis (Table 5), the strength of evidence was moderate to low for all non-significant meta-analyses due to inconsistency and imprecision. The significantly smaller reduction in GI of green tea mouthwash compared to chlorhexidine mouthwash was supported by evidence of moderate strength. For the treatment of periodontitis (Table 6) the strength of evidence was low to very low in all instances due to bias and inconsistency.

**Table 5:** Summary of findings table according to the GRADE approach for meta-analyses on gingivitis patients

Outcome Studies (patients)	Anticipated absolute effects (95% CI)				
	Control group^a^	Difference in experimental group	Quality of the evidence (GRADE)^b^	What happens in experimental group	Comment
**Green tea vs. control / placebo**					
PI 4 studies (137 patients)	-0.48	0.55 smaller (1.17 smaller to 0.07 greater)	⨁⨁◯◯ Low^c, d^ due to inconsistency, imprecision	Little to no difference in PI	Based on an SMD for PI_SL_ / PI_QH_ of -1.90 (95% CI -4.05 to 0.25); back-translated to PI_SL_ using an average control SD of 0.29
GI 3 studies (77 patients)	-0.34	0.26 smaller (0.56 smaller to 0.04 greater)	⨁⨁◯◯ Low^c, d^ due to inconsistency, imprecision	Little to no difference in GI	-
**Green tea vs. chlorhexidine**					
PI 6 studies (209 patients)	-0.73	0.14 smaller (0.31 smaller to 0.03 greater)	⨁⨁◯◯ Low^c, d^ due to inconsistency, imprecision	Little to no difference in PI	Based on an SMD for PI_SL_ / PI_QH_ of -0.40 (95% CI -0.88 to 0.09); back-translated to PI_SL_ using an average control SD of 0.35
GI 3 studies (150 patients)	-0.89	0.08 greater (0.01 to 0.14 greater)	⨁⨁⨁◯ Moderate^d^ due to imprecision	Might lead to smaller GI reduction than chlorhexidine	-
**Turmeric vs. chlorhexidine**					
PI 4 studies (240 patients)	-2.48	0.11 greater (0.20 smaller to 0.42 greater)	⨁⨁◯◯ Low^c, d^ due to inconsistency, imprecision	Little to no difference in PI	Based on an SMD for PI_SL_ / PI_QH_ of 0.28 (95% CI -0.53 to 1.10); back-translated to PI_SL_ using an average control SD of 0.38
GI 4 studies (240 patients)	-0.90	0.03 smaller (0.10 smaller to 0.04 greater)	⨁⨁⨁◯ Moderate^d^ due to imprecision	Little to no difference in GI	-

Population: adolescent or adult patients with plaque-induced gingivitis; intervention: supplementation of standard treatment with green tea or turmeric mouthwash; comparison: standard treatment or use of chlorhexidine mouthwash; setting: university clinics (India, Iran, and United States of America)

^a^ Response in the control group is based on the response of included studies (or random-effects meta- analysis of the control response)

^b^ Starts from “high”

^c^ Signs of inconsistency, as potential effects include considerable reductions to considerable increases

^d^ Imprecision due to the limited number of small studies with limited sample sizes

CI, confidence interval; GI, gingival index; GRADE, Grades of Recommendation, Assessment, Development, and Evaluation; PI, plaque index; QH, Quigley-Hein index; SD, standard deviation; SL, Silness and Löe index; SMD, standardised mean difference

**Table 6:** Summary of findings table according to the GRADE approach for meta-analyses on periodontitis patients

Outcome Studies (patients)	Anticipated absolute effects (95% CI)				
	Control group^a^	Difference in experimental group	Quality of the evidence (GRADE)^b^	What happens in experimental group	Comment
**Green tea vs. control / placebo**					
GI 3 studies (148 patients)	-1.04	0.53 smaller (0.05 to 1.01 smaller)	⨁⨁◯◯ Low^c, d^ due to bias, inconsistency	Might lead to reduced GI	-
Probing depth 4 studies (154 patients)	-1.18 mm	0.79 mm smaller (0.29 to 1.29 mm smaller)	⨁◯◯◯ Very low^d, e^ due to bias, inconsistency	Might lead to reduced probing depth	-
**Turmeric vs. control / placebo**					
PI 4 studies (230 patients)	-0.62	0.23 smaller (0.80 smaller to 0.35 greater)	⨁⨁◯◯ Low^c, f^ due to bias, inconsistency	Little to no difference in PI	Based on an SMD for PI_SL_ / PI_QH_ of -0.49 (95% CI -1.74 to 0.75); back-translated to PI_SL_ using an average control SD of 0.46
GI 3 studies (170 patients)	-0.63	0.23 smaller (0.63 smaller to 0.17 greater)	⨁◯◯◯ Very low^d, e^ due to bias, inconsistency	Little to no difference in GI	-
Probing depth 4 studies (230 patients)	-1.03	0.28 mm smaller (0.77 mm smaller to 0.21 mm greater)	⨁⨁◯◯ Low^c, f^ due to bias, inconsistency	Little to no difference in probing depth	-
**Turmeric vs. chlorhexidine**					
PI 2 studies (110 patients)	-0.55	0.03 greater (0.04 smaller to 0.11 greater)	⨁⨁◯◯ Low^c, f^ due to bias, inconsistency	Little to no difference in PI	Based on an SMD for PI_SL_ / PI_QH_ of 0.07 (95% CI -0.09 to 0.23); back-translated to PI_SL_ using an average control SD of 0.47
GI 2 studies (110 patients)	-0.35	0.02 greater (0.11 smaller to 0.15 greater)	⨁⨁◯◯ Low^c, f^ due to bias, inconsistency	Little to no difference in GI	-
Probing depth 2 studies (110 patients)	-0.85	0.14 mm greater (0.06 mm smaller to 0.34 mm greater)	⨁⨁◯◯ Low^c, f^ due to bias, inconsistency	Little to no difference in probing depth	-

Population: adolescent or adult patients with periodontitis; intervention: supplementation of standard treatment with green tea or turmeric (mouthwash or local administration); comparison: standard treatment or use of chlorhexidine mouthwash; setting: university clinics (India, Japan, and Thailand)

^a^ Response in the control group is based on the response of included studies (or random-effects meta- analysis of the control response)

^b^ Starts from “high”

^c^ Downgraded by one due to risk of bias

^d^ Signs of inconsistency, as potential effects span from very large reduction to minimally relevant reduction

^e^ Downgraded by two due to severe risk of bias

^f^ Signs of inconsistency, as potential effects include considerable reductions to considerable increases

CI, confidence interval; GI, gingival index; GRADE, Grades of Recommendation, Assessment, Development, and Evaluation; PI, plaque index; QH, Quigley-Hein index; SD, standard deviation; SL, Silness and Löe index; SMD, standardised mean difference

## Discussion

### Evidence in context

The present systematic review summarizes data from 19 randomized trials on 814 human patients using green tea extract or turmeric for the treatment of gingivitis and periodontitis. Evidence from included studies indicated that neither green tea nor turmeric conferred significant benefits for the treatment of gingivitis. As far as periodontitis treatment is concerned, the use of green tea was associated with significant benefits in terms of improved periodontal parameters, while no such indications were seen for turmeric.

Treatment of plaque-induced gingivitis was not found to benefit from supplemental administration of either green tea extract or turmeric through mouthwashes. On the contrary, green tea mouthwash was associated with smaller GI reduction than chlorhexidine mouthwash (Table 3), the efficacy of which has long since been proven [26–28]. Green tea extracts in mouthwashes have previously been shown to be effective in reducing plaque scores [29].

Periodontitis treatment was enhanced through the topical application of green tea extract in terms of improved probing depth, gingival inflammation, and plaque control (Table 6). Catechins in general possess anti-oxidant, anti-carcinogenic, anti-inflammatory, and anti-microbial properties [30–32] and green tea catechins have been shown to inhibit the growth of certain periodontal pathogens like P. gingivalis [33, 34] and T. forsythia [35]. Green tea catechins have been shown to also possess anti-collagenase properties, which might aid in preventing bone resorption and lead to greater probing depth reduction [36]. They might also reduce potential periodontal breakdown through their inhibition of the cysteine proteinases of P. gingivalis and the protein tyrosine phosphatase of P. intermedia [37, 38], which are considered periodontal virulence factors.

In this systematic review, studies evaluating the effects of a professional application of components of superfoods on the response to periodontal treatment were critically appraised. Several decisions had to be taken at the beginning of this review, as no universally accepted definition exists for so-called “superfoods” [39]. The term “superfood” suggests usually foods rich in macro- and micronutrients, which might have positive effects on human health or might contribute to disease prevention and / or treatment [40]. However, the concept of superfoods in terms of food or beverages consumed through a patient’s nutrition limits the scope of experimental intervention in a controlled clinical setting, where we can ensure that the application is made by trained clinicians under controlled conditions. For this reason, this review focused post hoc on the experimental administration of two selected superfoods (green tea and turmeric) in the controlled environment of human intervention trials, where a concise administration protocol can be employed and replicated.

### Limitations

The present review has several limitations. First, only studies in English or German were included and this might introduce language bias [41]. The majority of included studies originated from specific parts of the world (mostly from India) and therefore their results need to be confirmed by independent studies from different regions. Additionally, large variation existed in used administration protocols, and it was not possible to identify the most efficient protocol in each case. Several included studies had limited sample sizes and this leads to evidence of imprecision in the GRADE approach [23]. Finally, several included studies were found to be in high risk of bias, which has been shown to sometimes influence their effect estimates [42]. Future studies should actively compare different concentrations or administration methods, include an adequate sample size based on appropriate a priori calculations, follow contemporary guidelines for their conduct / reporting [18, 43], have an a priori registration / protocol [44], and ensure open data provision to increase transparency [45].

## Conclusion

Evidence from randomized trials on humans failed to find any significant benefits from the use of mouthwashes containing green tea extract or turmeric for the treatment of gingivitis. Local supplementation with green tea extract during periodontitis treatment was found to be associated with improved outcomes in terms of probing depth, gingival index, and plaque index. On the other hand, local supplementation with turmeric was not found to be associated with improved outcomes of periodontitis treatment. However, no strong clinical recommendation can be made, due to the small number and high risk of bias of existing studies and additional research is needed.

## Electronic supplementary material

Below is the link to the electronic supplementary material.

Supplementary Material 1

## Data Availability

The dataset for this study is available (10.5281/zenodo.13629830).
