# Supplementary material for: Weighing up the potential of “superfoods” compounds of green tea or turmeric as adjuncts in comparison to established therapeutical approaches for periodontal disease
Source: Clin Oral Investig. 2025 Jan 14;29(1):61. doi: 10.1007/s00784-024-06122-2 (PMC11732890; doi:10.1007/s00784-024-06122-2)
Supplement: Supplementary file 1 — Supplementary Material 1 [file 784_2024_6122_MOESM1_ESM.docx]

**Supplementary material**

**Weighing up the potential of “superfoods” compounds of green tea or turmeric as adjuncts in comparison to established therapeutical approaches for periodontal disease**

**Appendix 1.** Supplementary review details.

Additional method details

- When results were provided as data before treatment and data after treatment a Pre/Post correlation of 0.75 (back-calculated from studies providing raw data) was used to calculate treatment-induced changes, according to the methods described in the Cochrane Handbook.
- Data provided as medians and interquartile ranges were transformed to means (median~mean) and standard deviations, according to the method of Wan et al. (DOI: 10.1186/1471-2288-14-135).
- Results of pairwise meta-analyses were presented in contour-enhanced forest plots illustrating the magnitude of observed effects. This helps to assess the precision, heterogeneity, and clinical relevance of observed effects. For Standardized Mean Differences (SMD), effects of 0.2, 0.5, and 0.8 were used as cut-off points to denote small, moderate, large, and very large effects. For Mean Differences (MD) we identified the average Standard Deviation (SD) at baseline of the control group in each comparison among meta-analyzed studies and used effects of 0.5, 1.0, and 2.0 SDs as cut-off points to denote small, moderate, large, and very large effects.
- Reporting biases were planned to be assessed through the drawing of contour-enhanced funnel plots and formally with the Thompson test. As, however, no meta-analysis of at least 10 studies was performed, no reporting bias analysis was ultimately not conducted.
- Post hoc decision: following suggestion by a reviewer, we conducted a sensitivity analysis of meta-analysis comparing active agents to control / placebo groups, to assess potential differences between the comparison group (no treatment / placebo versus standard treatment).

**Appendix 2.** Superfood categories

| **berries** | **sprouts** | **fruits** | **citruses** | **seeds** | **spices** | **onion vegetables** | **root vegetables** | **legumes** | **vegetables** | **seaweeds** | **grains** | **nuts** | **meat** | **herbs** | **milks** | **fish** | **teas** | **resins** | **honeys** |
| --- | --- | --- | --- | --- | --- | --- | --- | --- | --- | --- | --- | --- | --- | --- | --- | --- | --- | --- | --- |
| blueberry | bamboo shoots | noni | lime | cocoa, -powder, -nibs, -butter | pepper | garlic | yacon | soy, -bean | carrot | chlorella | barley grass | pistachio | chicken meat | moringa | milk | fish | green tea | pollen | honey |
| acai berry | cress | avocado | lemon | coffee, -powder, -beans | saffron | onion | ginger | mesquite | broccoli | seaweed | dinkel grass | cedar nut | beef | nettle | soy milk | salmon | black tea | propolis | chestnut honey |
| aronia | alfalfa sprouts | jackfruit | grapefruit | chia seed | cinnamon | leek | maca | kidney bean | spinach | spirulina, -tabs, -powder | quinoa | hazelnut | veal | dandelion | almond milk |  | matcha green tea |  | fir honey |
| mulberry | mung bean sprouts | plum | mandarin | linseed |  | wild garlic | galangal | pea | Brussel sprout | irish moss |  | walnut | pork meat | rosehip | oat milk |  |  |  | clover honey |
| strawberry |  | ananas | orange | fled seed, psyllium, psyllium husk |  |  | ginseng | lentils | eggplant |  |  | cashew | mutton | pearl barley, icelandicus, fever moss, blood lung moss | rice milk |  |  |  | manuka honey |
| raspberry |  | camu camu fruit | lychee | black caraway |  |  | Jerusalem artichockes | peanut | amaranth |  |  | Brazil nut | lamb | verbena | cannabis milk |  |  |  | dandelion honey |
| blackberry |  | apricot | bergamot | cannabis seed |  |  | celery |  | chili |  |  | pecan | rabbit meat | chard | lupine milk |  |  |  | acacia honey |
| acerola |  | lucuma | clementine |  |  |  | parsnip |  | sweet peppers, pepperoni |  |  | macademia |  |  | pea milk |  |  |  | thyme honey |
| cranberry |  | date fruit | satsuma |  |  |  | potato |  | zucchini, courgette |  |  | nutsedge |  |  | cashew milk |  |  |  | lavender honey |
| goji berry |  | baobab | minneola, ugli |  |  |  | radish |  | cucumber |  |  | pine nut |  |  | dinkel milk |  |  |  | robinia honey |
| barberry |  | fig |  |  |  |  | beetroot |  | cantaloupe |  |  | chestnut |  |  | macademia milk |  |  |  | sunflower honey |
| blackcurrant |  | guarana |  |  |  |  | curcuma, turmeric |  | watermelon |  |  | ginkgo |  |  | coconut milk |  |  |  | fennel honey |
| currant |  | khaki |  |  |  |  |  |  | cauliflower |  |  |  |  |  | hazelnut milk |  |  |  | oak honey |
| gooseberry |  | pomegranate |  |  |  |  |  |  | romanesco |  |  |  |  |  |  |  |  |  | linden honey |
| elderberry |  | coconut |  |  |  |  |  |  | kale |  |  |  |  |  |  |  |  |  | orange blossom honey |
| rowan |  | cherry |  |  |  |  |  |  | cabbage |  |  |  |  |  |  |  |  |  | rape honey |
| sea buckthorn |  | sour cherry |  |  |  |  |  |  | artichocke |  |  |  |  |  |  |  |  |  | eucalyptus honey |
|  |  | mango |  |  |  |  |  |  | savoy |  |  |  |  |  |  |  |  |  | pine honey |
|  |  | papaya |  |  |  |  |  |  | arugula |  |  |  |  |  |  |  |  |  | forest honey |
|  |  | apple |  |  |  |  |  |  | sorrel |  |  |  |  |  |  |  |  |  | heather honey |
|  |  | olive |  |  |  |  |  |  |  |  |  |  |  |  |  |  |  |  |  |
|  |  | guava |  |  |  |  |  |  |  |  |  |  |  |  |  |  |  |  |  |
|  |  | quince |  |  |  |  |  |  |  |  |  |  |  |  |  |  |  |  |  |
|  |  | peach |  |  |  |  |  |  |  |  |  |  |  |  |  |  |  |  |  |
|  |  | starfruit |  |  |  |  |  |  |  |  |  |  |  |  |  |  |  |  |  |
|  |  | wild plum |  |  |  |  |  |  |  |  |  |  |  |  |  |  |  |  |  |
|  |  | tamarind |  |  |  |  |  |  |  |  |  |  |  |  |  |  |  |  |  |
|  |  | passionfruit |  |  |  |  |  |  |  |  |  |  |  |  |  |  |  |  |  |
|  |  | kiwi |  |  |  |  |  |  |  |  |  |  |  |  |  |  |  |  |  |
|  |  | mirabelle |  |  |  |  |  |  |  |  |  |  |  |  |  |  |  |  |  |

**Appendix 3.** List of excluded studies.

| **Author** | **Year** | **Title** | **Reason for Exclusion** |
| --- | --- | --- | --- |
| Abascal, K. | 2001 | Herbs for treating periodontal disease | No RCT |
| Abraham, Sajith | 2005 | Evaluation of the inhibitory effect of triphala on PMN-type matrix metalloproteinase (MMP-9) | In vitro |
| Agarwal, Garima | 2012 | Evaluation of chemical composition and efficacy of Chinese propolis extract on Porphyromonas gingivalis and Aggregatibacter actinomycetemcomitans: An in vitro study | In vitro |
| Agarwal, R. | 2013 | Essential oils in dentistry-an update | In vitro |
| Akhmetova, D. M. | 2008 |  | Not in English |
| Akhtar, J. | 2011 | A review on phytochemical and pharmacological investigations of miswak (Salvadora persica Linn) | No RCT |
| Allaker, R. P. | 2009 | Novel anti-microbial therapies for dental plaque-related diseases | No RCT |
| Al-Maweri S et al. 2021. | 2021 | Curcumin mouthwashes versus chlorhexidine in controlling plaque and gingivitis: a systematic review and meta-analysis | No RCT |
| Alvarez Hernandez, Maria | 2006 | Use of olive oil in the preparation of a product for oral hygiene for eliminating or reducing bacterial plaque and/or bacteria in the mouth | No RCT |
| Amin | 2019 |  | No RCT |
| Amurdhavani, B. S. | 2015 | Benefits of green tea in dentistry-a review | No RCT |
| Anderson, Michael R. | 2003 | Oral hygiene powder composition and method | No RCT |
| Anonymous, | 1996 | Natural compounds fight oral pathogens | No RCT |
| Arab, H. | 2011 | Review of the therapeutic effects of Camellia sinensis (green tea) on oral and periodontal health | No RCT |
| Araghizadeh, A. | 2013 | Inhibitory activity of green tea (Camellia sinensis) extract on some clinically isolated cariogenic and periodontopathic bacteria | In vitro |
| Aslani, Abolfazl | 2016 | Design, formulation, and physicochemical evaluation of periodontal propolis mucoadhesive gel | In vitro |
| Atwa, Al-Dany A. | 2014 | Effect of honey in preventing gingivitis and dental caries in patients undergoing orthodontic treatment | Insuff. Clinical parameters |
| Awadalla, H. I. | 2011 | A pilot study of the role of green tea use on oral health | Intervention too short |
| Bachrach, G. | 2011 | Garlic allicin as a potential agent for controlling oral pathogens | In vitro |
| Bagan, J. V. | 2012 | Cytological changes in the oral mucosa after use of a mouth rinse with alcohol: A prospective double blind control study | Not related to topic |
| Bairy, I. | 2002 | Evaluation of antibacterial activity of Mangifera indica on anaerobic dental microglora based on in vivo studies | Insuff. Clinical parameters |
| Bakri, I. M. | 2005 | Inhibitory effect of garlic extract on oral bacteria | In vitro |
| Balci, N. | 2015 | Anti-inflammatory agents in mouth-rinses for periodontal treatment | No RCT |
| Bardají, D. K. R. | 2016 | Copaifera reticulata oleoresin: Chemical characterization and antibacterial properties against oral pathogens | In vitro |
| Barretto, E. S. | 2014 | Treatment options from our winged friends: A review on propolis and its potential applications in dentistry | No RCT |
| Bedran, T. B. L. | 2015 | Black tea extract and its theaflavin derivatives inhibit the growth of periodontopathogens and modulate interleukin-8 and β-defensin secretion in oral epithelial cells | In vitro |
| Below, H. | 2017 | Measurements of chlorhexidine, p-chloroaniline, and p-chloronitrobenzene in saliva after mouth wash before and after operation with 0.2% chlorhexidine digluconate in maxillofacial surgery: a randomised controlled trial | Insuff. Clinical parameters |
| Ben Lagha, A. | 2015 | Wild Blueberry (Vaccinium angustifolium Ait.) Polyphenols Target Fusobacterium nucleatum and the Host Inflammatory Response: Potential Innovative Molecules for Treating Periodontal Diseases | In vitro |
| Ben Lagha, A. | 2017 | Black tea theaflavins attenuate Porphyromonas gingivalis virulence properties, modulate gingival keratinocyte tight junction integrity and exert anti-inflammatory activity | In vitro |
| Ben Lagha, Amel | 2017 | Tea polyphenols inhibit the growth and virulence properties of Fusobacterium nucleatum | In vitro |
| Ben Lagha, Amel | 2018 | Dual action of highbush blueberry proanthocyanidins on Aggregatibacter actinomycetemcomitans and the host inflammatory response | In vitro |
| Benly, P. | 2016 |  | No RCT |
| Bersan, S. M. F. | 2014 | Action of essential oils from Brazilian native and exotic medicinal species on oral biofilms | In vitro |
| Bhadbhade, S. J. | 2011 | The antiplaque efficacy of pomegranate mouthrinse | Intervention too short |
| Bharath, Nagaraj | 2015 | Determination of antibacterial activity of green coffee bean extract on periodontogenic bacteria like Porphyromonas gingivalis, Prevotella intermedia, Fusobacterium nucleatum and Aggregatibacter actinomycetemcomitans: An in vitro study | In vitro |
| Bhattarai, Govinda | 2017 | Anti-inflammatory, anti-osteoclastic, and antioxidant activities of genistein protect against alveolar bone loss and periodontal tissue degradation in a mouse model of periodontitis | Animal study |
| Bhavana, S. | 2017 | A randomized clinical trial to assess and compare the antimicrobial activity of plants of lythraceae family with hiora mouth washes in subjects with chronic periodontitis - "Unveiling the unseen effects" | Intervention too short |
| Bhavikatti, S. K. | 2015 | Triphala: Envisioning its role in dentistry | No RCT |
| Bhuvaneswari, P. | 2014 | Antioxidants in oral healthcare | No RCT |
| Biswas, G. | 2015 | Effect of grape products on oral health- A Review | No RCT |
| Bodet, C. | 2006 | Anti-inflammatory activity of a high-molecular-weight cranberry fraction on macrophages stimulated by lipopolysaccharides from periodontopathogens | In vitro |
| Bodet, C. | 2007 | Cranberry components inhibit interleukin-6, interleukin-8, and prostaglandin E2 production by lipopolysaccharide-activated gingival fibroblasts | In vitro |
| Bodet, C. | 2008 | Potential oral health benefits of cranberry | No RCT |
| Bodet, Charles | 2006 | Inhibition of periodontopathogen-derived proteolytic enzymes by a high-molecular-weight fraction isolated from cranberry | In vitro |
| Bohora, A. A. | 2017 | Good Bugs vs Bad Bugs: Evaluation of Inhibitory Effect of Selected Probiotics against Enterococcus faecalis | In vitro |
| Bommareddy, Praveen K. | 2013 | Antibacterial and anti-proliferative activity of Isolated Fractions of Aqueous extract from the rhubarb stem | No RCT |
| Botushanov, P. I. | 2001 |  | No RCT |
| Bruschi, Marcos L. | 2007 | Semisolid systems containing propolis for the treatment of periodontal disease: in vitro release kinetics, syringeability, rheological, textural, and mucoadhesive properties | In vitro |
| Bruschi, Marcos Luciano | 2008 | Precursor system of liquid crystalline phase containing propolis microparticles for the treatment of periodontal disease: development and characterization | In vitro |
| Chandakavathe, B. N., D. K. Deshpande, P. V. Swamy and S. B. Dhadde (2018). | 2018 | "Assessment of Toothpaste Formulations Containing Turmeric and Neem Extract for Prevention of Dental Caries and Periodontal Diseases." | In Vitro |
| Chatterjee, Anirban | 2011 | Probiotics in periodontal health and disease | No RCT |
| Chaturvedi, T. P. | 2009 | Uses of turmeric in dentistry: An update | No RCT |
| Chee, B. | 2016 | Omega-3 fatty acids as an adjunct for periodontal therapy-a review | No RCT |
| Chezhian, N. | 2017 |  | No RCT |
| Chun, J. W. | 2003 | Killing effects and inhibition of biofilm formation on human teeth against oral microorganisms by "green tea gargle" for dental care | Not related to topic |
| Coates, Alison | 2012 | Fish oil supplementation as adjunct therapy for periodontitis | No RCT |
| Cortelli, Jose Roberto | 2012 | Validation of the anti-bacteremic efficacy of an essential oil rinse in a Brazilian population: a cross-over study | No RCT |
| [Ctri (2018).](http://www.who.int/trialsearch/Trial2.aspx?TrialID=CTRI/2018/01/011237) | 2018 | Comparison of efficacy of 2 different mouthrinses. | No RCT |
| [Ctri (2018).](http://www.who.int/trialsearch/Trial2.aspx?TrialID=CTRI/2018/05/014002) | 2018 | Effect of Green tea and commercially available Mouthwash on gum diseases. | No RCT |
| [Ctri (2019).](http://www.who.int/trialsearch/Trial2.aspx?TrialID=CTRI/2019/04/018697) | 2019 | "Local Drug Delivery for the treatment of Periodontal Diseases." | No RCT |
| [Ctri (2019).](http://www.who.int/trialsearch/Trial2.aspx?TrialID=CTRI/2019/05/019336) | 2019 | Antimicrobial efficacy of White Tea and Green Tea mouthrinse against Dental Plaque, gingivitis and Salivary Streptococcus mutans. | No RCT |
| [Ctri (2020).](http://www.who.int/trialsearch/Trial2.aspx?TrialID=CTRI/2020/01/022707) | 2020 | Evaluation of zinc oxide -turmeric extract dressing after periodontal surgery in chronic periodontitis patients. | No RCT |
| Dabholkar, C. S. | 2016 | Comparative evaluation of antimicrobial activity of pomegranate-containing mouthwash against oral-biofilm forming organisms: An invitro microbial study | In vitro |
| de Medeiros, Annie Karoline Bezerra | 2016 | Inhibitory effect of cranberry extract on periodontopathogenic biofilm: An integrative review | No RCT |
| de Sousa, F. O. | 2012 | Effect of zein on biodegradable inserts for the delivery of tetracycline within periodontal pockets | In vitro |
| Dejoie, S. | 2016 | Anti-inflammatory activity of a Carica papaya leaf extract used to prevent occasional gingivitis | No RCT |
| Desjardins, J. | 2012 | Anthocyanin-rich black currant extract and cyanidin-3-O-glucoside have cytoprotective and anti-inflammatory properties | In vitro |
| Devaraj, S. D. | 2014 | Curcumin- Pharmacological actions and its role in dentistry | No RCT |
| Dewi, R. S. | 2017 | Effect of 12.5% virgin coconut oil on porphyromonas gingivalis and treponema denticola bacterial colonization | Intervention too short |
| Dhinahar, S. | 2011 | Role of botanicals as antimicrobial agents in management of dental infections - A review | No RCT |
| Didry, N. | 1998 | Antimicrobial activity of aerial parts of Drosera peltata Smith on oral bacteria | In vitro |
| DiSilvestro, Robert A. | 2009 |  | Insuff. Clinical data |
| Dodington | 2015 |  | No RCT |
| Dua, K. | 2015 | Antimicrobial efficacy of extemporaneously prepared herbal mouthwashes | In vitro |
| El Sharkawy | 2016 |  | Invalid population |
| Eley, B. M. | 1999 | Antibacterial agents in the control of supragingival plaque--a review | No RCT |
| El-Sharkawy, H. | 2010 | Adjunctive treatment of chronic periodontitis with daily dietary supplementation with omega-3 fatty acids and low-dose aspirin | Not related to topic |
| El-Shinnawi, U. | 2015 | Actions of adjunctive nutritional antioxidants in periodontitis and prevalent systemic inflammatory diseases | No RCT |
| Elumalai, M. | 2014 | Herbs used in dentistry | No RCT |
| Feghali, K. | 2012 | Cranberry proanthocyanidins: Natural weapons against periodontal diseases | No RCT |
| Feres, Magda | 2005 | In vitro antimicrobial activity of plant extracts and propolis in saliva samples of healthy and periodontally-involved subjects | In vitro |
| Fine, D. H. | 2010 |  | Insuff. Clinical data |
| Forouzanfar F et al. 2020 | 2020 | Curcumin for the Management of Periodontal Diseases: A Review | No RCT |
| Forouzanfar, Ali | 2016 |  | Not in English |
| Gangadhar, V. | 2011 | Correlation between leptin and the health of the gingiva: A predictor of medical risk | Not related to topic |
| Gao, Q. | 2018 |  | Insuff. Clinical data |
| Gartenmann S et al. 2020 | 2020 | The Effect of Green Tea on plaque and gingival inflammation: A systematic review | No RCT |
| Gaultier, F. | 2005 | Lupinus albus, a novel vegetable extract with metalloproteinase inhibitory properties: A potential periodontal therapy | In vitro |
| Gaur, S. | 2014 | Green tea: A novel functional food for the oral health of older adults | No RCT |
| Gebara, Elaine C. E. | 2002 | Propolis antimicrobial activity against periodontopathic bacteria | No RCT |
| Ghaibie, N. | 2016 |  | Intervention too short |
| Gonzalez, O. A. | 2013 | Antibacterial effects of blackberry extract target periodontopathogens | In vitro |
| Grenier, D. | 2015 | Dual action of myricetin on Porphyromonas gingivalis and the inflammatory response of host cells: A promising therapeutic molecule for periodontal diseases | In vitro |
| Gromova, L. E. | 2016 | PHARMACOLOGICAL CORRECTION OF CHRONIC GENERALIZED CATARRHAL GINGIVITIS WITH A GARLIC PREPARATION | Insuff. Clinical parameters |
| Gu, Ying | 2013 | 4-methoxycarbonyl curcumin: a unique inhibitor of both inflammatory mediators and periodontal inflammation | Animal study |
| Gursoy, U. K. | 2009 | Anti-biofilm properties of Satureja hortensis L. essential oil against periodontal pathogens | In vitro |
| H, R. R. | 2017 | Formulation of thermoreversible gel of cranberry juice concentrate: Evaluation, biocompatibility studies and its antimicrobial activity against periodontal pathogens | In vitro |
| Habiboallah, Ghanbari | 2008 | Histological evaluation of Curcuma longa-ghee formulation and hyaluronic acid on gingival healing in dog | Animal study |
| Hamazaki, K. | 2006 | Fish oil reduces tooth loss mainly through its anti-inflammatory effects? | Insuff. Clinical parameters |
| Hamilton, J. A. | 2017 | Atherosclerosis, Periodontal Disease, and Treatment with Resolvins | No RCT |
| Horie, Norio | 2014 | Anti-inflammatory potential of Rikkosan based on IL-1beta network through macrophages to oral tissue cells | In vitro |
| Hosadurga, Rajesh Ramesh | 2014 | Evaluation of the efficacy of 2% curcumin gel in the treatment of experimental periodontitis | Animal study |
| Hosadurga, Rajesh Ramesh | 2015 | Evaluation of the efficacy of 2% Ocimum sanctum gel in the treatment of experimental periodontitis | Animal study |
| Hsaine, S. | 2017 | Evaluation of antibacterial activity of essential oil of Cinnamomum zeylanicum, Eugenia caryophyllata, and Rosmarinus officinalis against Streptococcus oralis | In vitro |
| Ishikado, A. | 2010 | Human trial of liposomal lactoferrin supplementation for periodontal disease | Not related to topic |
| Iwasaki, Masanori | 2010 | Longitudinal relationship between dietary omega-3 fatty acids and periodontal disease | Not related to topic |
| Izui, S. | 2016 | Antibacterial Activity of Curcumin Against Periodontopathic Bacteria | In vitro |
| Jagan Rao, N. | 2012 | Role of phytotherapy in gingivitis: A review | No RCT |
| Jalaluddin, M., I. Jayanti, I. M. Gowdar, R. Roshan, R. R. Varkey and A. Thirutheri (2019). | 2019 | "Antimicrobial Activity of Curcuma longa L. Extract on Periodontal Pathogens." | In Vitro |
| Jangid, K. | 2014 | Achievable therapeutic effects of myristica fragrans (NUTMEG) on periodontitis a short review | No RCT |
| Jangid, K. | 2016 | Anti-protease activity of Myristica fragrans compared to doxycycline on periodontal tissues: An ex-vivo study | In vitro |
| Johnston, B. D. | 2013 | Use of dietary supplements in patients seeking treatment at a periodontal clinic | Insuff. Clinical parameters |
| Josino Soares, D. | 2014 | Pitanga (Eugenia uniflora L.) fruit juice and two major constituents thereof exhibit anti-inflammatory properties in human gingival and oral gum epithelial cells | In vitro |
| Jovito | 2016 |  | Intervention too short |
| Kanatas, A. | 2008 | RE: Visavadia BG, Honeysett J, Danford MH. Manuka honey dressing: An effective treatment for chronic wound infections. Br J Oral Maxillofac Surg. 2006 Nov 17; [Epub ahead of print] | No RCT |
| Karygianni, Lamprini | 2015 | Natural Antimicrobials and Oral Microorganisms: A Systematic Review on Herbal Interventions for the Eradication of Multispecies Oral Biofilms | No RCT |
| Katsura, H. | 2001 | In vitro antimicrobial activities of bakuchiol against oral microorganisms | In vitro |
| Kemoli, A. M. | 2001 | Antimicrobial and buffer capacity of crude extracts of chewing sticks (Miswaki) from Kenya | In vitro |
| Kim, J. H. | 1997 | Anti-bacterial action of onion (Allium cepa L.) extracts against oral pathogenic bacteria | In vitro |
| Kim, Se Eun | 2017 | Efficacy of horse chestnut leaf extract ALH-L1005 as a matrix metalloproteinase inhibitor in ligature-induced periodontitis in canine | Animal study |
| Kobayashi, H. | 2005 | Suppression of lipopolysaccharide-induced cytokine production of gingival fibroblasts by a soybean, Kunitz trypsin inhibitor | In vitro |
| Kukreja, B. J. | 2012 | Herbal mouthwashes - A gift of nature | No RCT |
| Kumar, G. | 2013 | Emerging trends of herbal care in dentistry | No RCT |
| Lazar, Veronica | 2016 | Periodontitis and Periodontal Disease - Innovative Strategies for Reversing the Chronic Infectious and Inflammatory Condition by Natural Products | No RCT |
| Lee, Kyung-Yeol | 2013 | Synergistic effect of fucoidan with antibiotics against oral pathogenic bacteria | In vitro |
| Lewis, C. | 2002 | Fighting gum disease: how to keep your teeth | No RCT |
| Li Y et al. 2021 | 2021 | Curcumin: A review of experimental studies and mechanisms related to periodontitis treatment | No RCT |
| Lowe, G. | 2003 | Total tooth loss and prevalent cardiovascular disease in men and women: Possible roles of citrus fruit consumption, vitamin C, and inflammatory and thrombotic variables | No RCT |
| Maestre-Vera, J. R. | 2004 | Treatment options in odontogenic infection | No RCT |
| Magro-Filho, O. | 1994 | Topical effect of propolis in the repair of sulcoplasties by the modified Kazanjian technique. Cytological and clinical evaluation | Insuff. Clinical parameters |
| Malaiappan et al. 2020 | 2020 | Effect of pomegranate green tea mouthwash in orogranulocyte migratory rate in gingivitis patients | insuff. Clinical parameters |
| Manika, | 2015 | Curcumin - A solid gold in medicine and dentistry | No RCT |
| Maruyama, Takayuki | 2011 | Supplementation of green tea catechins in dentifrices suppresses gingival oxidative stress and periodontal inflammation | Animal study |
| Mathur A et al. 2018 | 2018 | Efficacy of green tea-based mouthwashes on dental plaque and gingival inflammation: A systematic review and meta-analysis | No RCT |
| Miyake, Yoshiaki | 2011 | Isolation and extraction of antimicrobial substances against oral bacteria from lemon peel | In vitro |
| Mohan, M. | 2018 | The role of green tea in oral health - A review | No RCT |
| Morawiec, T | 2013 |  | Invalid population |
| Muniz, F. W. | 2015 | The impact of antioxidant agents complimentary to periodontal therapy on oxidative stress and periodontal outcomes: A systematic review | No RCT |
| Nagpal, Monika | 2013 | Role of curcumin in systemic and oral health: An overview | No RCT |
| Nakagawa, Taneaki | 1997 |  | Not in English |
| Naqvi, A. Z. | 2014 | Docosahexaenoic Acid and Periodontitis in Adults: A Randomized Controlled Trial | Not related to topic |
| [Nct (2019).](https://clinicaltrials.gov/show/NCT03790904) | 2019 | Anti Plaque Efficacy of Salvadora Persica L. and Green Tea Mouthwash. | No RCT |
| [Nct (2020).](https://clinicaltrials.gov/show/NCT04355416) | 2020 | Clinical and Anti-inflammatory Effect of Curcumin Oral Gel as Adjuncts in Treatment of Periodontal Pocket. | No RCT |
| Ng, N. | 2014 | Coffee consumption and periodontal disease in males | No RCT |
| Nittayananta, Wipawee | 2018 | Oral spray containing plant-derived compounds is effective against common oral pathogens | In vitro |
| Nivetha, R. | 2014 | Effects of natural products on oral health: A review | No RCT |
| Ofek, Itzhak | 2012 | Functional Foods: Towards Improving Oral Health | No RCT |
| Onakpoya, I. | 2016 |  | No RCT |
| Pashaev, Ch A. | 1994 |  | Not in English |
| Patel, V. K. | 1983 |  | No RCT |
| Pawar Vinita, A. | 2013 | Formulation and evaluation of dental gel containing essential oil of coriander against oral pathogens | No RCT |
| Peedikavil | 2015 |  | No RCT |
| Peng, J. | 1991 |  | Not in English |
| Penmetsa, G. S. | 2017 | Comparative evaluation of multiflower honey, jamun honey and chlorhexidine gluconate gel (0.2%) on clinical levels of dental plaque: One week randomized controlled clinical trial | Intervention too short |
| Pinto, S. A. Holanda | 2008 | Anti-inflammatory effect of alpha, beta-Amyrin, a pentacyclic triterpene from Protium heptaphyllum in rat model of acute periodontitis | Animal study |
| Polak, David | 2013 | Protective Potential of Non-Dialyzable Material Fraction of Cranberry Juice on the Virulence of P. gingivalis and F. nucleatum Mixed Infection | Animal study |
| Pooja, S. | 2016 | Antioxidants and its role in periodontitis - A short review | No RCT |
| Prasad, Divyashree | 2014 | Punica granatum: A review on its potential role in treating periodontal disease | No RCT |
| Preetha, S. | 2015 | Effect of various irrigants on dental biofilm: A review | No RCT |
| Prithi, R. | 2014 | Static effects of fruits on periodontitis | No RCT |
| Ramachandran, S. | 2012 | Garlic and onion in dentistry | No RCT |
| Ramaiah, V. V., S. U. Tareen, A. J. Sayed, M. M. Al-Mutairi and Z. A. Alsuwaydani (2020). | 2020 | Evaluation of efficacy of green tea neem extract, frezyderm and rexidine mouthwash on plaque induced gingivitis. | insuff. Clinical parameters |
| Ramamurthy, J. | 2011 | Pharmacological aspects of tea tree oil (TTO) and its role in dentistry - A comprehensive review | No RCT |
| Ramasamy, C. | 2015 | Potential natural antioxidants: Adjuvant effect of green tea polyphenols in periodontal infections | No RCT |
| Ravi, K. | 2014 | Psidium guajava: A review on its potential as an adjunct in treating periodontal disease | No RCT |
| Renuka, S. | 2017 | Comparison in benefits of herbal mouthwashes with chlorhexidine mouthwash: A review | No RCT |
| Renvert, S. | 1981 | Healing after treatment of periodontal intraosseous defects. II. Effect of citric acid conditioning of the root surface | Not related to topic |
| Reyhaneh, Sariri | 2011 | Essential oil of tea leaves provides oral hygiene and prevents oral bacterial growth | No RCT |
| Rizaev, J. O. | 2005 |  | Not in English |
| Rojanapanthu, Pleumchitt | 2006 | Andrographis paniculata gel as an adjunct in the treatment of periodontitis | No RCT |
| Rosas-Piñón, Y. | 2012 | Ethnobotanical survey and antibacterial activity of plants used in the Altiplane region of Mexico for the treatment of oral cavity infections | No RCT |
| Rosenstein, E. D. | 2003 | Pilot study of dietary fatty acid supplementation in the treatment of adult periodontitis | Not related to topic |
| Safiaghdam H et al. 2018 | 2018 | Medicinal plants for gingivitis: A review of clinical trials | No RCT |
| Saitawee, D. | 2018 | Photodynamic therapy of Curcuma longa extract stimulated with blue light against Aggregatibacter actinomycetemcomitans | In vitro |
| Salehi B et al. 2019 | 2019 | Plant-derived bioactives in oral mucosal lesions: A key emphasis to Curcumin, Lycopene, chamomile, aloe Vera, green tea and coffee properties | No RCT |
| Santi S et al. 2021 | 2021 | Effect of herbal mouthrinses on dental plaque formation and gingival inflammation: A systematic review | No RCT |
| Santos, Paulo Sergio da Silva | 2008 | Use of oral rinse with enzymatic system in patients totally dependent in the intensive care unit | Insuff. Clinical parameters |
| Santos, V. R. | 2009 |  | Insuff. Clinical data |
| Sardana, D. | 2013 | Role of propolis in dentistry: Review of the literature | No RCT |
| Sathiyawathie, S. | 2015 | Nature's recipe for oral health | No RCT |
| Satthanakul, P. | 2015 | Antimicrobial effect of lemongrass oil against oral malodour micro-organisms and the pilot study of safety and efficacy of lemongrass mouthrinse on oral malodour | Not related to topic |
| Sculley, D. V. | 2014 | Periodontal disease: modulation of the inflammatory cascade by dietary n-3 polyunsaturated fatty acids | No RCT |
| Sekar, M. | 2016 | Formulation, evaluation and antibacterial properties of novel Polyherbal toothpaste for oral care | in vitro |
| Shabbir, Ambreen | 2016 | Propolis, A Hope for the Future in Treating Resistant Periodontal Pathogens | in vitro |
| Shafiei, Z. | 2012 | Antibacterial activity of Myristica fragrans against oral pathogens | in vitro |
| Shahakbari, R. | 2014 |  | Invalid population |
| Shekar, B. R. C. | 2015 | Herbal extracts in oral health care - A review of the current scenario and its future needs | No RCT |
| Sindhuja S et al. 2019 | 2019 | Clinical efficacy and anti-inflammatory property of curcumin in periodontal disease: A systematic review | No RCT |
| Singh, J. M. | 2007 | Can Jua be a weapon in combating oral diseases? | No RCT |
| Skaba, Dariusz | 2013 |  | Insuff. Clinical data |
| Soltani, R. | 2014 | Evaluation of the effect of green tea extract on the prevention of gingival bleeding after posterior mandibular teeth extraction: A randomized controlled trial | Insuff. Clinical parameters |
| Sonmez, S. | 2005 | The effect of bee propolis on oral pathogens and human gingival fibroblasts | in vitro |
| Spratt, D. A. | 2012 | Evaluation of plant and fungal extracts for their potential antigingivitis and anticaries activity | in vitro |
| Srinath, J. | 2014 | Application of spices in dentistry- A literature review | No RCT |
| Stipcevic, Tamara | 2006 | Use of rhamnolipids in wound healing, treatment and prevention of gum disease and periodontal regeneration | No RCT |
| Stoyell, K. A. | 2016 | Clinical efficacy of turmeric use in gingivitis: A comprehensive review | No RCT |
| Svatun, B. | 1987 | The influence of a dentifrice containing a zinc salt and a nonionic antimicrobial agent on the maintenance of gingival health | Not related to topic |
| Tamanai-Shacoori, Z. | 2014 | Silver-zeolite combined to polyphenol-rich extracts of ascophyllum nodosum potential active role in prevention of periodontal diseases | in vitro |
| Thangavelu, A. | 2017 | Ancient seed for modern cure - Pomegranate review of therapeutic applications in periodontics | No RCT |
| Thomas, K. E. | 2014 | Honey in the treatment of periodontitis | No RCT |
| Van Der Velden, U. | 2011 | Micronutritional approaches to periodontal therapy | No RCT |
| Varoni, E. M. | 2012 | Plant polyphenols and oral health: Old phytochemicals for new fields | No RCT |
| Vasanti, S. | 2009 | Antimicrobial activity of clove essential oil against clinical dental isolates | in vitro |
| Venkateswara, Babu | 2011 | Green tea extract for periodontal health | No RCT |
| Vieira, D. R. P. | 2014 | Plant species used in dental diseases: Ethnopharmacology aspects and antimicrobial activity evaluation | in vitro |
| Weber, Charles | 2005 | Eliminate infection (abscess) in teeth with cashew nuts | No RCT |
| Weinberg, A. | 1998 | Epithelial antimicrobial peptides: Review and significance for oral applications | No RCT |
| Weiss, Ervin I. | 1998 | Inhibiting interspecies coaggregation of plaque bacteria with a cranberry juice constituent | in vitro |
| Wiȩckiewicz, Wł | 2013 | Does propolis help to maintain oral health? | No RCT |
| Wu, Christine D. | 2009 | Grape products and oral health | No RCT |
| Yim, N. | 2010 | The antimicrobial activity of compounds from the leaf and stem of Vitis amurensis against two oral pathogens | in vitro |
| Zanyk, L. | 1990 | Fighting plaque on the home front | No RCT |
| Zeidán-Chuliá, F. | 2013 | MMP-REDOX/NO interplay in periodontitis and its inhibition with satureja hortensis L. Essential Oil | in vitro |
| Zhao, L. | 2013 | Antibacterial, antiadherence, antiprotease, and anti-inflammatory activities of various tea extracts: Potential benefits for periodontal diseases | in vitro |
| [No authors] | 2010 | Green tea and oral health examined in study | No RCT |

_

**Appendix 4.** Overview of included studies in qualitative synthesis.

|  | **APPLICATION / INTAKE** | | | | | | |  |
| --- | --- | --- | --- | --- | --- | --- | --- | --- |
| **SUPERFOOD** | **Mouthwash** | **Local delivery**  **(chip or gel)** | **SRP** | **Dentifrice** | **Gel (topical)** | **Oral consumption** | **Supplement** | **TOTAL** |
| **Green Tea** | Balappanavar 2013  Lauten 2005  Priya 2017  Yaghini 2019  Andhare 2022  Deshpande 2022  Sharma 2023 | Chava 2013  Hirasawa 2002  Rattanasuwan 2016  Nagate 2020 | Wang 2021 | Hrishi 2016 |  | Chopra 2016 | Tripathi 2019 | 15 |
| **Curcuma** | Divya 2017  Mali 2012  Waghmare 2011  Sarkar 2023 | Anuradha 2015  Singh 2018  Kaur 2019  Guru 2020  Singh 2021 |  |  | Singh 2015 |  |  | 10 |
| **Pomegranate** | Batista 2014 | Sastravaha 2005 | Abbulais 2015 |  | Prakash 2017  Somu 2011 |  |  | 5 |
| **Propolis** | Neumann 1986 | Sanghani 2014 | Gebara 2003  Coutinho 2012 | Tanasiewicz 2012 |  |  |  | 5 |
| **Polyherbal Mixture** | Mahyari 2016 |  | Pistorius 2003 |  |  |  |  | 2 |
| **Ayurvedic** | Gupta 2013 |  |  |  |  |  |  | 1 |
| **Cinnamon** | Gupta 2015 |  |  |  |  |  |  | 1 |
| **Papaya** |  | Kharaeva 2016 |  |  |  |  |  | 1 |
| **Spirulina** |  | Mahendra 2013 |  |  |  |  |  | 1 |
| **Manuka Honey** | Singhal 2018 |  |  |  |  | English 2004 |  | 2 |
| **Lemongrass** | Dany 2015 | Warad 2013 |  |  |  |  |  | 2 |
| **Barberry** |  |  |  | Makarem 2005 |  |  |  | 1 |
| **Eggplant** | Diab 2011 |  |  |  |  |  |  | 1 |
| **Misc** |  |  |  |  |  | Chapple 2012 |  | 1 |
| **Dietary advice** |  |  |  |  | Zare 2014 |  |  | 1 |
| **TOTAL** | 19 | 14 | 5 | 3 | 4 | 3 | 1 | 49 |

SRP, scaling and root planing

**Appendix 5.** Detailed risk of bias assessment of included randomized trials with the Cochrane ROB 2 tool.

| Domain | **Andhare 2022** | **Anuradha 2015** | **Balappanavar 2013** | **Chava 2013** | **Deshpande 2021** | **Divya 2017** | **Guru 2020** | **Hirasawa 2002** | **Kaur 2019** | **Lauten 2005** | **Mali 2012** | **Nagate 2020** | **Priya 2017** | **Rattanasuwan 2016** | **Sarkar 2023** | **Sharma 2023** | **Singh 2018** | **Singh** | **Waghmare 2011** | **Yaghini 2019** |
| --- | --- | --- | --- | --- | --- | --- | --- | --- | --- | --- | --- | --- | --- | --- | --- | --- | --- | --- | --- | --- |
|  |  |  |  |  |  |  |  |  |  |  |  |  |  |  |  |  |  | **2021** |  |  |
| **Domain 1: RoB arising from the randomization process (Selection bias)** |  |  |  |  |  |  |  |  |  |  |  |  |  |  |  |  |  |  |  |  |
| 1.1. Was the allocation sequence random? | **Y** | **Y** | **Y** | **Y** | **Y** | **Y** | **Y** | **Y** | **Y** | **Y** | **N** | **Y** | **Y** | **Y** | **Y** | **Y** | **Y** | **Y** | **Y** | **Y** |
| 1.2. Was the allocation sequence concealed until participants were enrolled and assigned to interventions? | **Y** | **Y** | **?** | **Y** | **Y** | **Y** | **Y** | **Y** | **Y** | **Y** | **Y** | **Y** | **Y** | **Y** | **Y** | **Y** | **Y** | **Y** | **Y** | **Y** |
| 1.3. Did baseline differences between intervention groups suggest a problem with the randomization process? | **N** | **N** | **N** | **N** | **N** | **N** | **N** | **N** | **N** | **N** | **PY** | **N** | **N** | **N** | **N** | **N** | **N** | **N** | **N** | **N** |
| **Domain 1 judgement** | **low** | **low** | **low** | **low** | **low** | **low** | **low** | **low** | **low** | **low** | **some concerns** | **low** | **low** | **low** | **low** | **low** | **low** | **low** | **low** | **low** |
|  |  |  |  |  |  |  |  |  |  |  |  |  |  |  |  |  |  |  |  |  |
| **Domain 2: RoB due to deviations from the intended interventions (effect of assignment to intervention) (Selection bias)** |  |  |  |  |  |  |  |  |  |  |  |  |  |  |  |  |  |  |  |  |
| 2.1. Were participants aware of their assigned intervention during the trial? | **PY** | **PY** | **Y** | **N** | **N** | **N** | **N** | **N** | **N** | **N** | **N** | **N** | **N** | **N** | **N** | **N** | **N** | **Y** | **N** | **N** |
| 2.2. Were carers and people delivering the interventions aware of participants’ assigned intervention during the trial? | **N** | **Y** | **Y** | **Y** | **N** | **N** | **Y** | **Y** | **N** | **N** | **?** | **Y** | **N** | **N** | **N** | **N** | **Y** | **Y** | **Y** | **N** |
| 2.3. If Y/PY/NI to 2.1. or 2.2.: Were there deviations from the intended intervention that arose because of the experimental context? | **N** | **N** | **N** | **N** | **N** | **-** | **N** | **N** | **N** | **-** | **N** | **N** | **-** | **-** | **N** | **N** | **N** | **N** | **N** | **-** |
| 2.4. If Y/PY to 2.3.: Were these deviations from intended intervention balanced between groups? | **-** | **-** | **-** | **-** | **-** | **-** | **-** | **-** | **-** | **-** | **-** | **-** | **-** | **-** | **-** | **-** | **-** | **-** | **-** | **-** |
| 2.5. If N/PN/NI to 2.4.: Were these deviations likely to have affected the outcome? | **-** | **-** | **-** | **-** | **-** | **-** | **-** | **-** | **-** | **-** | **-** | **-** | **-** | **-** | **-** | **-** | **-** | **-** | **-** | **-** |
| 2.6. Was an appropriate analysis used to estimate the effect of assignment to intervention? | **Y** | **N** | **Y** | **N** | **N** | **N** | **Y** | **N** | **Y** | **N** | **N** | **N** | **N** | **N** | **N** | **N** | **N** | **N** | **Y** | **N** |
| 2.7. If N/PN/NI to 2.6.: Was there potential for a substantial impact (on the result) of the failure to analyze participants in the group to which they were randomized? | **N** | **N** | **-** | **N** | **N** | **N** | **-** | **Y** | **-** | **N** | **Y** | **N** | **N** | **N** | **N** | **N** | **N** | **N** | **-** | **N** |
| **Domain 2 judgement** | **low** | **high** | **some concerns** | **some concerns** | **low** | **low** | **some concerns** | **some concerns** | **low** | **low** | **some concerns** | **some concerns** | **low** | **low** | **low** | **low** | **high** | **high** | **some concerns** | **low** |
|  |  |  |  |  |  |  |  |  |  |  |  |  |  |  |  |  |  |  |  |  |
| **Domain 3: RoB due to missing outcome data (Performance bias)** |  |  |  |  |  |  |  |  |  |  |  |  |  |  |  |  |  |  |  |  |
| 3.1. Were data for this outcome available for all, or nearly all, participants randomized? | **Y** | **Y** | **Y** | **Y** | **Y** | **Y** | **Y** | **Y** | **Y** | **Y** | **Y** | **Y** | **Y** | **PY** | **Y** | **Y** | **Y** | **Y** | **Y** | **Y** |
| 3.2. If N/PN/NI to 3.1.: Is there evidence that the result was not biased by missing outcome data? | **-** | **-** | **-** | **-** | **-** | **-** | **-** | **-** | **-** | **-** | **-** | **-** | **-** | **-** | **-** | **-** | **-** | **-** | **-** | **-** |
| 3.3. If N/PN to 3.2.: Could missingness in the outcome depend on its true value? | **-** | **-** | **-** | **-** | **-** | **-** | **-** | **-** | **-** | **-** | **-** | **-** | **-** | **-** | **-** | **-** | **-** | **-** | **-** | **-** |
| 3.4. If Y/PY/NI to 3.3.: Is it likely that missingness in the outcome depended on its true value? | **-** | **-** | **-** | **-** | **-** | **-** | **-** | **-** | **-** | **-** | **-** | **-** | **-** | **-** | **-** | **-** | **-** | **-** | **-** | **-** |
| **Domain 3 judgement** | **low** | **low** | **low** | **low** | **low** | **low** | **low** | **low** | **low** | **low** | **low** | **low** | **low** | **low** | **low** | **low** | **low** | **low** | **low** | **low** |
|  |  |  |  |  |  |  |  |  |  |  |  |  |  |  |  |  |  |  |  |  |
| **Domain 4: RoB in measurement of the outcome (Attrition bias)** |  |  |  |  |  |  |  |  |  |  |  |  |  |  |  |  |  |  |  |  |
| 4.1. Was the method of measuring the outcome inappropriate? | **N** | **N** | **N** | **N** | **N** | **N** | **N** | **N** | **N** | **N** | **N** | **N** | **N** | **N** | **N** | **N** | **N** | **N** | **N** | **N** |
| 4.2. Could measurement or ascertainment of the outcome have differed between intervention groups? | **N** | **?** | **N** | **N** | **N** | **N** | **N** | **PN** | **N** | **N** | **?** | **N** | **N** | **N** | **N** | **N** | **?** | **N** | **?** | **N** |
| 4.3. If N/PN/NI to 4.1. and 4.2.: Were outcome assessors aware of the intervention received by study participants? | **N** | **Y** | **Y** | **Y** | **?** | **Y** | **N** | **Y** | **N** | **N** | **?** | **Y** | **N** | **N** | **?** | **?** | **Y** | **Y** | **Y** | **N** |
| 4.4. If Y/PY/NI to 4.3.: Could assessment of the outcome have been influenced by knowledge of intervention received? | **N** | **Y** | **N** | **Y** | **N** | **N** | **?** | **Y** | **-** | **-** | **?** | **Y** | **-** | **-** | **-** | **N** | **N** | **N** | **N** | **-** |
| 4.5. If Y/PY/NI to 4.4.: Is it likely that assessment of the outcome was influenced by knowledge of intervention received? | **-** | **Y** | **-** | **Y** | **N** | **-** | **-** | **Y** | **-** | **-** | **?** | **Y** | **-** | **-** | **-** | **-** | **-** | **-** | **-** | **-** |
| **Domain 4 judgement** | **low** | **high** | **low** | **some concerns** | **low** | **low** | **low** | **high** | **low** | **low** | **high** | **high** | **low** | **low** | **Some concerns** | **low** | **some concerns** | **Some concerns** | **low** | **low** |
|  |  |  |  |  |  |  |  |  |  |  |  |  |  |  |  |  |  |  |  |  |
| **Domain 5: RoB in selection of the reported result (Reporting bias)** |  |  |  |  |  |  |  |  |  |  |  |  |  |  |  |  |  |  |  |  |
| 5.1. Were the data that produced this result analyzed in accordance with a pre-specified analysis plan that was finalized before unblinded outcome data were available for analysis? | **Y** | **N** | **Y** | **N** | **Y** | **N** | **Y** | **N** | **Y** | **N** | **N** | **N** | **N** | **Y** | **N** | **Y** | **Y** | **Y** | **Y** | **N** |
| Is the numerical result being assessed likely to have been selected, on the basis of the results, from… |  |  |  |  |  |  |  |  |  |  |  |  |  |  |  |  |  |  |  |  |
| 5.2. …multiple outcome measurements (*eg* scales, definitions, time points) within the outcome domain? | **N** | **N** | **N** | **N** | **N** | **N** | **N** | **N** | **N** | **N** | **N** | **N** | **N** | **N** | **N** | **N** | **N** | **N** | **N** | **N** |
| 5.3. …multiple analysis of the data? | **N** | **N** | **N** | **N** | **N** | **N** | **N** | **N** | **N** | **N** | **N** | **N** | **N** | **N** | **N** | **N** | **N** | **N** | **N** | **N** |
| **Domain 5 judgement** | **low** | **low** | **low** | **low** | **low** | **low** | **low** | **low** | **low** | **low** | **low** | **low** | **low** | **low** | **low** | **low** | **low** | **low** | **low** | **low** |
| **Overall RoB judgement** | **+** | **-** | **+** | **-** | **+** | **+** | **?** | **-** | **+** | **+** | **-** | **-** | **+** | **+** | **+** | **+** | **-** | **-** | **?** | **+** |

RoB, risk of bias.

**Appendix 6.** Meta-analysis on the effect of green tea extract supplementation versus control for the treatment of gingivitis in terms of plaque index.

Adm, administration mode; CI, confidence interval; MW, mouthwash; n1/2, patients in experimental / control group; M1/2, mean in experimental / control group; SD1/2, standard deviation in experimental / control group; SMD, standardized mean difference.

**Appendix 7.** Meta-analysis on the effect of green tea extract supplementation versus control for the treatment of gingivitis in terms of gingival index.

Adm, administration mode; MD, mean difference; CI, confidence interval; MW, mouthwash; n1/2, patients in experimental / control group; M1/2, mean in experimental / control group; SD1/2, standard deviation in experimental / control group.

**Appendix 8.** Meta-analysis on the effect of green tea extract supplementation versus chlorhexidine for the treatment of gingivitis in terms of plaque index.

Adm, administration mode; CI, confidence interval; MW, mouthwash; n1/2, patients in experimental / control group; M1/2, mean in experimental / control group; SD1/2, standard deviation in experimental / control group; SMD, standardized mean difference.

**Appendix 9.** Meta-analysis on the effect of turmeric supplementation versus chlorhexidine for the treatment of gingivitis in terms of plaque index.

Adm, administration mode; CI, confidence interval; MW, mouthwash; n1/2, patients in experimental / control group; M1/2, mean in experimental / control group; SD1/2, standard deviation in experimental / control group; SMD, standardized mean difference.

**Appendix 10.** Meta-analysis on the effect of turmeric supplementation versus chlorhexidine for the treatment of gingivitis in terms of gingival index.

Adm, administration mode; MD, mean difference; CI, confidence interval; MW, mouthwash; n1/2, patients in experimental / control group; M1/2, mean in experimental / control group; SD1/2, standard deviation in experimental / control group.

**Appendix 11.** Meta-analysis on the effect of turmeric supplementation versus control for the treatment of periodontitis in terms of plaque index.

Adm, administration mode; CI, confidence interval; MW, mouthwash; n1/2, patients in experimental / control group; M1/2, mean in experimental / control group; SD1/2, standard deviation in experimental / control group; SMD, standardized mean difference.

**Appendix 12.** Meta-analysis on the effect of turmeric supplementation versus control for the treatment of periodontitis in terms of gingival index.

Adm, administration mode; MD, mean difference; CI, confidence interval; MW, mouthwash; n1/2, patients in experimental / control group; M1/2, mean in experimental / control group; SD1/2, standard deviation in experimental / control group.

**Appendix 13.** Meta-analysis on the effect of turmeric supplementation versus control for the treatment of periodontitis in terms of probing depth.

Adm, administration mode; MD, mean difference; CI, confidence interval; MW, mouthwash; n1/2, patients in experimental / control group; M1/2, mean in experimental / control group; SD1/2, standard deviation in experimental / control group.

**Appendix 14.** Meta-analysis on the effect of turmeric supplementation versus chlorhexidine for the treatment of periodontitis in terms of plaque index.

Adm, administration mode; MD, mean difference; CI, confidence interval; MW, mouthwash; n1/2, patients in experimental / control group; M1/2, mean in experimental / control group; SD1/2, standard deviation in experimental / control group.

**Appendix 15.** Meta-analysis on the effect of turmeric supplementation versus chlorhexidine for the treatment of periodontitis in terms of gingival index.

Adm, administration mode; MD, mean difference; CI, confidence interval; MW, mouthwash; n1/2, patients in experimental / control group; M1/2, mean in experimental / control group; SD1/2, standard deviation in experimental / control group.

**Appendix 16.** Meta-analysis on the effect of turmeric supplementation versus chlorhexidine for the treatment of periodontitis in terms of probing depth.

Adm, administration mode; MD, mean difference; CI, confidence interval; MW, mouthwash; n1/2, patients in experimental / control group; M1/2, mean in experimental / control group; SD1/2, standard deviation in experimental / control group.

**Appendix 17.** Sensitivity analysis of meta-analyses assessing green tea, according to whether this was compared to control / placebo or standard treatment.

|  |  |  | **Placebo** | |  | **SRP** | |  |  |
| --- | --- | --- | --- | --- | --- | --- | --- | --- | --- |
| **Intervention** | **Outcome** |  | **Studies** | **Effect (95% CI)** |  | **Studies** | **Effect (95% CI)** |  | **P for differences** |
| Green tea | Plaque index_SL/QH_ |  | 3 | SMD -1.77 (-4.79, 1.25) |  | 2 | SMD -2.28 (-2.89, -1.68) |  | 0.74 |
| Green tea | Gingival index |  | 2 | MD -0.26 (-0.81, 0.29) |  | 1 | MD -0.24 (-0.33, -0.15) |  | 0.94 |

CI, confidence interval; QH, Quigley-Hein index; MD, mean difference; n, studies; SL, Silness and Löe index; SMD, standardized mean difference; SRP, scaling / root planing.
